# "The Hospital" Nursing Mirror

**Published:** 1900-10-27

**Authors:** 


"The Hospital" Nursing Mirror.

Being the Nursing Section of "The Hospital."

[Contributions for this Section of "The Hospital" should be addressed to the Editor, "The Hospital" Nursing Mirror, 28 & 29 Southampton Street, Strand, London, W.C.]

### Notes on News from the Nursing World.

### THE QUEEN AND THE CUSHION.

The Queen has paid Nurse Palmer a great compliment. Having charge of several soldiers who came to the Military Hospital at Sandgate from Ladysmith, Miss Palmer conceived the ingenious idea of making up a cushion from pieces of the discarded clothing of the men. When she had finished it she sent it to Her Majesty, who graciously accepted it, and caused the following letter to be forwarded to the delighted nurse:—

"Balmoral Castle, October 12.

"Dear Madam,—The Queen desires me to thank you for the cushion, which, Her Majesty is interested to hear, you made yourself from the discarded clothing of the men who came to the Beach Rocks Hospital at Sandgate from Ladysmith, and which, at the request of the invalid soldiers, you have been good enough to send, through the Rev. E. Evans, for Her Majesty's acceptance.—Yours faithfully,

"F. W. Ponsonby."

### HONOURS FOR ARMY NURSES.

There is, happily, no danger that the soldiers who have fought so bravely for their country in South Africa will not be adequately rewarded. But we hope that the nurses will not be forgotten. If they have not been exposed to peril on the field of battle, they have run the gauntlet of dangers hardly less formidable. Some of them have literally died at the post of duty—victims of typhoid—others have suffered severely in health; and most, if not all, have undergone serious privations. It would be invidious, and would create endless heart-burning, to make selections, and the best way to meet the case would be to bestow a war medal on each of the nurses, British or Colonial, whose services have been appreciated by the authorities. The war correspondents, it appears, are to be given a medal, and the claims of the nurses to the honour are decidedly stronger. The decoration would not be costly, and it would have the admirable effect of stimulating enthusiasm. No red tapeism ought, for a moment, to stand in the way of the award.

### RETURNING HOME.

Invalided and other nurses are now returning home from South Africa. The "Britannic," which is on its way to England, includes among the "sick and wounded" sisters E. Nixon, C. Bainbridge, and M. E. Warmington, of New Zealand. There are also seven nurses, namely:—Sisters McCall, Anderson, Monson, Tucker, Pryde, Shore, and Rogers on board, but these are on duty during the voyage. Sisters Sands, Bankes, Tarbuck and Driver arrived in the "Montrose" last week. In consequence of the decrease of serious cases at the Imperial Yeomanry Hospital, Sisters Higgs, Hodge, Penrose, Powell, and Rolleston are leaving for England.

### A COSTUME COMPETITION AT DEELFONTEIN.

There has been a costume competition at Deelfontein Hospital, in which several of the nurses took part. They appear to have acquitted themselves with success, for their costumes are said to have been in all cases ingenious, tasteful and charming, while some were strikingly beautiful. The Geisha costume of Sister Beedie, the Gipsy Fortune-teller of Sister Mustard, and the Sepsis of Sister Lancaster, were awarded by Lady Chesham, who acted as judge, equal first prizes.

### SISTER M. C. ANDERSON, R.R.C.

It was announced some time ago in our columns that the Queen had conferred the decoration of the Royal Red Cross upon Miss May Christina Anderson, nursing sister in charge of the Colonial Hospital, Suva, Fiji; and an English resident in Fiji sends us some interesting details of her career. Miss Anderson entered the Government Hospital, Fiji, early in 1894 as a probationer, and is the first nurse who has received a full training on up-to-date lines in that colony. She served her first year under the matroncy of Miss Webster-Wedderburn, formerly of the Nightingale Home, at St. Thomas's Hospital, and afterwards under the direction of Miss Louisa Beale, of the London Hospital and the Prince Alfred Hospital, Sydney; and being for a time the only nurse in training she enjoyed the unique advantage of receiving a full course of lectures and practical teaching all to herself, from the staff of three medical officers and a matron. After passing the Government examination with honours, Miss Anderson was appointed staff nurse, and, two years later became the sister-in-charge—a matron being dispensed with—and she is now assisting in the training of probationers herself. She was commended by the Secretary of State for the Colonies in 1895, when quite a junior, for "devotion and endurance;" and the Governor of Fiji presented a silver chatelaine to her as a mark of appreciation of her services. She always took a special interest in the patients annually sent in to the hospital from the ships of war on the station; and in 1897 nursed a number of complicated cases of tropical enteric fever contracted in Samoa on board H.M.S. "Lizard." Last year, when the disturbances took place between Mata'afa's tribesmen and the combined British and American squadrons under Captain Sturdee and Admiral Krantz, a number of our sick and wounded officers and bluejackets were transferred, as opportunity offered, from the fighting line at Apia to the Government hospital at Fiji. It is more especially in recognition of Miss Anderson's devotion and success in personally nursing these cases, assisted by her mother and by Nurse Austin, that the Queen has conferred upon Sister Anderson the much-prized Cross. She is one of the few nursing sisters, if not the only one, enrolled in a Volunteer force, being a member of the Fiji Volunteer Force, where she renders valuable aid in the Ambulance and First Aid classes.

### AN ATTACK UPON THE NURSES AT GUY'S.

A London daily paper has permitted an anonymous scribbler to make a sweeping attack on the nurses at Guy's Hospital. This individual, who is described as "a correspondent writing from Hampshire," says:—

"My wife was an inmate for three weeks early in this year, and I consider that the nurses are most neglectful, often cruel, and put the patients to much needless suffering, striking the children, and disobeying the doctors' orders, opening windows close to a patient's head on foggy winter's nights in direct disobedience to the doctors' orders."

There may, of course, be nurses at Guy's, or any other hospital, who are not sufficiently attentive to their duties, but the possible faults of a few are no excuse for the indictment of a class. However, the allegations of the Hampshire correspondent can doubtless be tested, if he will come into the open and give chapter and verse for them. If he does not, the assumption will be that he has no grounds for assertions which, if false, are not only cruel but cowardly. We are surprised that the *Daily Mail* should have printed such an attack unsigned.

### THE NEW MATRON OF LIVERPOOL INFIRMARY.

An excellent appointment has been made by the Chairman and Committee of the Royal Infirmary, Liverpool. They have selected for the very important post of lady superintendent and matron, Miss Elizabeth Mary Jones, who for eleven years has been continuously engaged in the work of the hospital. Entering the Liverpool Nurses' Training School in connection with the Infirmary in 1889, after receiving two years' training at the Hospital for Sick Children, Pendlebury, Miss Jones was subsequently Sister of a Medical and Surgical Ward for five years, and night superintendent for eighteen months. She was then appointed Assistant Lady Superintendent and Matron, and has filled that office for four years. Her testimonials were of the highest character, including one from the retiring lady superintendent and matron, who wrote, "Should she be elected, I feel sure that the hospital would gain a most efficient officer and the nursing staff a wise and judicious head." But her simple record of work done was the best credential she could offer.

### A MATRON OF TWENTY-TWO.

The Holbeach Board of Guardians have a curious idea of their duty to the sick and suffering in their infirmary. Out of 62 applicants for the post of matron of the workhouse, they have chosen a young woman of twenty-two, because she is about to marry the master. The marriage was not even settled until the guardians considered the applications, and calling the master before the Board, inquired whether he was ready to get married at once. He confessed that he had not contemplated such haste, but as the Board stipulated for the wedding at a definite time, he consulted the lady, and announced that it could take place forthwith. On his side the master had intimated that if any other applicant receives the post of matron he should resign. The Holbeach Guardians are now described, not quite accurately, as "matchmakers," which may be amusing, but people in the nursing world who treat such matters seriously will want to know what possible qualifications the newly-elected matron can have for her duties. We should be sorry to interfere with prospective connubial bliss, but we must point out that an occurrence of this sort serves well to illustrate the difficulties which may sometimes surround the position of a trained superintendent nurse who, by the mere accident of a workhouse master's matrimonial leanings, may become the subordinate of a matron who may perchance not only be untrained but may be only twenty-two years of age.

### PROBATIONERS IN WORKHOUSES.

A very unsatisfactory state of things prevails at the Oldham Infirmary. At a meeting of the Board of Guardians the other day a letter was read from Dr. Young, the medical officer, protesting against the appointment of four probationary nurses without his prior approval. Dr. Young also said that the arrangements at the workhouse were not such as to conduce to the proper training of nurses. "There was no house surgeon, no regular instructor, and no examination such as was now generally recognised to be essential in the training of certified nurses." The guardians did not relish this plain speaking, and eventually it was decided, in the first place, not to take any notice of Dr. Young's letter, and in the second, that in future the nurses' certificates be signed by the chairman of the hospital committee, the nurses' superintendent, and the clerk of the Board. Dr. Young has no legal right to demand that probationers should not be admitted to the infirmary without his sanction, but if, as he alleges, there is no proper training, the certificates are of little value.

### OUR CHRISTMAS DISTRIBUTION.

Several of our readers having written to inquire the kind of contributions likely to be most acceptable at Christmas to the patients at the hospitals, we may state that warm vests for men and boys, warm underclothing for women, and warm night-gowns for children, are always particularly appreciated. It is perhaps advisable to repeat that the parcels should be addressed to "The Editor, The Hospital, 28 and 29 Southampton Street, Strand, London, W.C.," and should be inscribed "Clothing Distribution."

### OPENING OF A HANDSOME NURSING HOME AT LIVERPOOL.

The new central home for the Queen's nurses in Liverpool was formally opened by Mrs. William Rathbone last Friday. It is the gift of Mr. B. W. Levy and the David Lewis Trust to the city, and has cost about £10,000. An old building was acquired about a year ago, of red brick with freestone dressings, and the large new wing which has been added is in harmony with it. The old portion has been entirely rearranged to meet the requirements of the work for which it is intended. It contains a large dining-room, board room, nurses' and matron's sitting-rooms, about a dozen bedrooms, a room for appliances, which is also to be used as a lecture room, together with large kitchens and sculleries, &c.; also an isolation department consisting of a bedroom and bathroom, which is approached by a private door. This is for the use of any nurse who may contract an infectious disease. The wing which has been added contains ten bedrooms and the secretary's and matron's offices. In connection with the wing is a flat roof upon which is a picturesque little tea-room. Here, in summer, the nurses will be able to take tea and the fresh air under most pleasant conditions. The whole building has ample accommodation for a staff of twenty people. In the nurses' sitting-room is a fine grand piano, and there is a good library containing medical and other books. The electric light has been installed throughout the building. At the opening ceremony, which was attended by the Lord Mayor, several speeches were made, including one from Dr. Peile, the Master of St. Katherine's, who advises people in doubt about the work of the Queen's nurses, to go and ask the poor what they think about it. He predicted that "they would find that the poor regarded the nurses as angels sent by the Queen to go into their homes with light and comfort, such as they had never known before." And Dr. Peile says that he has seen the nurses "in all parts of their work."

### A QUEEN'S NURSE AT NORTON.

The people of Norton in the West Riding of Yorkshire are warned that they must not always rely upon the liberality of the most munificent subscriber to the nursing institution. Since it was founded in the year of the Queen's Diamond Jubilee, Colonel Legard, of Welham, has contributed the sum of £30 as salary for the nurse sent down from the Jubilee Institute. This is very generous of the Colonel, but we agree with a local contemporary that now the great services rendered by the nurse have been acknowledged and taken advantage of, it is only fair that other persons should come forward with their subscriptions, and assist in the good work being done amongst the poor. During the past year the nurse had in hand over 200 cases, and paid upwards of 2,500 visits, a sufficient reason why her salary should no longer be provided by one individual.

### STOCKPORT SICK POOR NURSING ASSOCIATION.

At the ninth annual meeting of the Stockport Sick Poor Nursing Association the report was adopted. Mention was made of the satisfactory results of the parade of the Trades and Labour Council in December last, which realised £48 5s.; of the increase of the nursing staff, and of the selection of a new home for the nurses. The last is described as the most important event in the Society's year, and the home itself is to be opened next month. It is intended to receive patients of limited means requiring surgical or medical treatment, but in the event of this new departure not being a success the deficiency will not come out of the charity—a very proper decision. On the other hand, if there is any profit on the private nursing, it will go to the support of the sick poor fund. The sick poor cannot therefore suffer in consequence of a laudable attempt to provide the necessities of the middle classes.

### NURSES' HOSTEL COMPANY.

The third annual report of the Nurses' Hostel Company shows that the receipts from the Hostel visitors amounted to £2,252, an increase of £146 over those of the previous year; and it is stated that the pressure on the accommodation has at times taxed the resources of the management to the utmost. The income exceeded the expenditure to the extent of £458, and one item in the latter of £145 was for decorative repairs, which will not appear again for some years. The directors recommended the payment of a dividend of 4 per cent., which, with £200 added to the reserve, leaves a balance of £17 13s. 8d. to carry forward to the next account. This was agreed to at the meeting of shareholders on Monday, and Miss Clegg and Mr. Lionel Earle, who retired by rotation, were re-elected directors.

### THE SUNDERLAND NURSES.

In the course of a speech delivered by Lady Londonderry in her capacity of President of the Sunderland District Nursing Association, she congratulated the managers on the success already achieved, but suggested that an endeavour should be made to interest workmen at the big works to subscribe, by promising them, on certain conditions, a representative on the Committee. It is true that the workmen contribute largely to the expenses of the Infirmary, but there can be no doubt that many of the men would sooner have their sick wives nursed at home. The fact that in less than three years the nurses of the Association have nursed 1,673 patients, paid nearly 53,000 visits, and attended 152 operations, appears sufficient evidence of its usefulness.

### AN OMISSION OF SEVEN WORDS.

Quite a sensation was caused in Cardiff last week by the announcement that the late Lord Bute's bequest of £20,000 to the Seamen's Hospital in that port was conditional on the sick being received and served by Sisters of some Roman Catholic order. Happily, there is no stipulation of the kind in the will. The words "if such an arrangement can be made" were left out in the terms of the document given to the Press, and, we need scarcely add, make all the difference.

### A WORKHOUSE INFIRMARY IN A NEW LIGHT.

At the meeting of the Isle of Wight Board of Guardians last week a letter was read from Nurse Shoebridge giving a month's notice of her intention to resign. One of the guardians referred to the constant changes in the staff, and said that it seemed to be a training home for officers, whereupon the Chairman rejoined, "A matrimonial office."

### NURSES AND WORKHOUSE DIETARIES.

The order as to dietaries in workhouses just issued by the Local Government Board contains the following provision of interest to nurses:—

> That, in a case of urgent necessity, the nurse in charge of a sick inmate may obtain from the master of the workhouse any food or stimulant which may be required, without waiting for the arrival of the medical officer; but the nurse is required to show the medical officer at his next visit the counterfoil of the requisition in order that he may endorse the same and give any directions in the matter. When the circumstances permit, the "Statim Requisition," as it is briefly termed, should be countersigned by the superintendent nurse.

### SHORT ITEMS.

At the National Hospital for the Paralysed and Epileptic the Harvest Thanksgiving Service took place in the chapel last Thursday evening, when the Bishop of Islington officiated, assisted by Rev. E. C. Bedford, Rector of St. George the Martyr, Holborn, and Rev. W. R. Greenhill, Chaplain of the Hospital. The decorations carried out by Sisters Lawrence, Hopkins, and Sussans were very effective, the flowers and foliage used being supplied from the gardens of the hospital Convalescent Home at East Finchley.—Dr. Boxall will lecture on "Midwifery Emergencies" at the Midwives' Institute and Trained Nurses' Club, 12 Buckingham Street, Strand, on Friday, October 26th, at 7.45. A limited number of tickets for non-members can be obtained fro[illegible] the Secretary.—The new matron of Lodge Moor Hospit[illegible] Sheffield, was trained at Brownlow Hill Infirmary, Liv[illegible]ool, and was afterwards Sister at the City Hospital, [illegible]effield.—The Joint Committee of the Women's University Settlement, Southwark, the National Union of Women Workers, and the Charity Organisation Society have arranged for a course of six lectures to be given by Mr. Thomas Mackay, the well known writer on Poor Law and kindred subjects, at the Church House, Westminster, on Fridays, at 11.30 A.M., beginning on October 26th. All particulars concerning the lectures may be obtained from the Hon. Secretary to the Lectures Committee, Mrs. G. F. Hill, 10 Kensington Mansions, Earl's Court, S.W.—The scheme for the erection of a new infirmary just accepted by the Richmond (Surrey) Board of Guardians, includes a nurses' home.—It has been decided to convert the present medical officer's quarters at the Suffolk Lunatic Asylum into rooms for the use of the nurses.—Nursing Sister Hoadley, A.N.S., who arrived on board the Hospital Ship "Dunera," is returning in the same vessel, which is in dock for ten days. This is her second voyage in the "Dunera," which came over in July with sick.—The first of the winter series of entertainments to the in-patients of the Cancer Hospital, Fulham Road, was given on Thursday evening last under the direction of Miss Constance E. Bromfield, and proved a very great success.

### Lectures on Nursing for Probationers.

By E. MACDOWEL COSGRAVE, M.D., Lecturer to the Dublin Metropolitan Technical School for Nurses.

### NO. XVIII.—BEVERAGES.

THE water necessary for life may be taken disguised in many ways: these various forms may be conveniently classed under the heading of "beverages."

One group of beverages in common use contains tea, coffee, and cocoa. These are non-alcoholic, and are stimulant and restorative. They are generally taken hot; this adds to their restorative effect. Although from the milk and sugar they contain they have some food value, generally these additions are only sufficient to give flavour. As they are all made with boiling water, they are safe drinks when the water-supply is contaminated.

*Tea.*—The ordinary black tea is made from leaves which, after picking, are left in heaps until a process of fermentation has taken place.

Tea is made by infusing the leaves by pouring boiling water upon them. The kettle should be filled with fresh cold water, and, as soon as the water boils, it should be poured over the leaves, previously placed in a scalded-out teapot. Water that has been boiled for any length of time has its air expelled, and does not make good tea.

Tea should not be allowed to "draw" too long. At the end of five minutes or so it will have exhausted the good of the leaves. If tea has to be kept for a late-comer it should not be allowed to stand on the leaves, but should be poured into another teapot or heated jug. Tea during "drawing" should not be placed on the hob, but under a cosy.

The restorative effect of tea is partly caused by the theine, which is practically identical with the caffeine of coffee, and partly by the warmth of the fluid. Tea contains tannin, which is commonly blamed as a cause of dyspepsia, and various devices have been adopted to exclude it; but it is harmless, and properly-made tea is unlikely to do harm unless it is taken without food, when it provokes the stomach to secrete gastric juice, although there is nothing to be digested; or when it is taken very hot after a meat meal, and then it is not the tea which does harm but the hot fluid, which kills the pepsin necessary for the digestion of meat.

In some cases where dyspepsia already exists tea-drinking does harm, as the large amount of hot fluid relaxes the already distended stomach, and leaves room for flatulence to collect.

Nervousness sometimes follows tea-drinking, but only in cases where it is drunk to force the brain to work beyond its natural powers, and without proper food being taken; as a result the nervous system becomes overstrained.

*Coffee.*—The best coffee is made from "berries" that have been freshly roasted and ground. To get the strength needs boiling, but this dissipates the aroma; the best compromise is to make an infusion, pour it off into a heated jug, then to boil the grounds and add this decoction to the infusion previously made.

Coffee should be made very strong, so as to bear diluting with plenty of hot milk without tasting weak; this makes coffee a useful beverage for those who cannot take solid food.

Coffee is a strong heart-stimulant, and invigorates without subsequent collapse.

Coffee is often adulterated. A simple test is to throw a little into a wineglass of water; if it is pure, it will float and will hardly tinge the water; if, however, chicory or dandelion be present, they will sink and colour the water red. Many people like the addition of a little chicory.

*Cocoa.*—This is a milder and less stimulating beverage than either tea or coffee, and contains more food; unfortunately it is less refreshing.

The seeds broken up are called cocoa-nibs; after prolonged boiling, and removal of the floating cocoa-butter, they yield a good beverage.

Prepared cocoas are often used; they contain less cocoa-butter, and are more quickly prepared than the nibs. In some (wrongly called "soluble") the cocoa is ground up with starch; with boiling water the starch forms a mucilage in which the fine particles of cocoa remain suspended.

Chocolate is cocoa ground up with sugar and flavoured with vanilla; it generally contains a little starch. Van Houten's cocoa is prepared from the nibs, some of the fat being removed; as it does not contain starch it forms a thin drink.

All preparations which have drugs, such as kola, added should be avoided.

Artificial mineral waters have as their basis water surcharged with carbonic acid gas. Soda water, potash water, and lithia water have those salts added, but not in the "lowering" amount often ascribed to them. Indeed, such mineral waters are practically no more lowering than an equal quantity of plain water.

"Sparklets" are useful in the sick room, as they enable milk, cold tea, coffee, home-made lemonade, &c., to be aërated.

Lemonade and ginger-ale contain in addition to the flavouring agents a considerable amount of sugar, and so are not such refreshing drinks; it is the sugar that causes the lasting froth.

Ginger-ale is a pleasant adjunct to a convalescent's meals; if too hot, it may be diluted with an equal quantity of soda water.

Home-made lemonade, made by pouring boiling water on a cut-up lemon (the white rind being removed) and a little sugar, forms a refreshing acid drink.

Apple tea, made by pouring boiling water on a cut-up apple and sweetening, also is refreshing. Sometimes it is better liked if made with a roast apple.

Barley water is a soothing demulcent drink, and is grateful to a sore mouth or throat. It ought to be infused like tea, and not boiled. For children it may be sweetened with sugar candy.

Oatmeal drink, made by boiling a little oatmeal and slightly sweetening, is both refreshing and sustaining; it is useful if hard work has to be done.

If a cooling drink with an aperient effect is required, tamarind whey may be given.

Of the natural mineral waters Apollinaris is the best as a beverage, and should always be used abroad if the water-supply is doubtful. It is a very pure water, and every precaution is taken in washing and filling the bottles so that it may not be contaminated.

In rheumatic cases Contrexéville water is used, and when the kidneys are not acting well Vichy is used.

Most of the other natural waters contain so much mineral matters that they are medicines rather than beverages.

### From Bloemfontein in a Hospital Train to Pretoria.

By an Army Nurse.

Writing from the Palace of Justice Hospital, Pretoria, September 26th, our correspondent says: Luck has attended us. Instead of being given work at base hospitals, six of our party were sent to Springfontein, and the rest to Bloemfontein; the former simply an encampment on the veldt, which left one wondering what there was to name before Tommy and his paraphernalia arrived. Those of us who were sent to Bloemfontein were temporarily quartered in the "Dames' Institute"—in peace time an industrial school for girls, but now commandeered for medical purposes.

### The Comicalities of Tent Life.

As the hospital accommodation was limited, six of us were housed in a marquee just outside the gates, and were initiated into the comicalities of tent life. The night was very windy and the canvas flapped about and provided more fresh air than we desired. The tent from the inside looked like an old-clothes shop with bonnets and towels, cloaks and undergarments pinned up to the canvas, and the picturesqueness of the whole was increased by the presence of a stray lamb and the hospital cat that found their way in in the morning. We had just two days in Bloemfontein and were then ordered to proceed by No. 3 hospital train to Pretoria. After various vicissitudes of bursting boxes, lost luggage, and, not least, conveyance in an ambulance waggon guaranteed to stimulate the most torpid liver, we boarded our train on Sunday evening.

### Travelling 50,000 Miles.

One of the Sisters in charge told us she had travelled in it eleven months and over 50,000 miles. The berths, arranged in double rows on each side with a passage-way about 3½ feet wide down the centre, were most comfortable, as we found by experience; the upper ones have small windows from which a splendid view of the country can be had.

Very few wounded now travel in the hospital trains, the cases being chiefly convalescent enterics. We found it far nicer than the mail train, having the run of two wards containing 40 out of the 96 on board. A water famine seems inseparable from railway travelling in South Africa—one realises then in a small way what horrible discomforts our soldiers have so cheerfully put up with—indeed, in conversation, almost every man will tell you he didn't mind the bullets so much, but that short rations, hard marches, and want of water were worse by far to bear.

### The Demand for Newspapers.

The men on lines of communications appealed most strongly to our feelings—the eagerness with which they asked for any newspapers and the gratitude for cigarettes, mufflers, note paper, biscuits, chocolate, or post cards showed plainly what luxuries these were. I should very much like to suggest to any others travelling to the front, who have brought small gifts for Tommy, to have them at hand for distribution *on the way*, as they cannot be more appreciated or acceptable at any hospital. A doctor at a small railway station on the route was bewailing the want of literature for his patients, and told us that books were even more valued than newspapers.

### A Possible Cause of Enteric.

We had some excitements by the way. At one place called Geneva we were within half an hour of the enemy; indeed, from the time of leaving Bloemfontein till the Vaal River was crossed, the enemy was more or less at hand. If we found it hard at times to realise that we were really in the presence of actual war, grim reminders were at hand. The skeletons and bodies of thousands of animals—horses, mules, sheep—in all stages of decomposition, testified to the terrible sacrifice of animal life which had been exacted, and which accounted easily for cases of enteric on the lines of communication. The country is remarkably uninteresting, contrasting strongly with the beauty of Karoo in the north of Cape Colony. Miles and miles of parched brown grass, interspersed with blacker patches of veldt fires, is all that one sees, and dotted all along the line, encampments of various sizes filled with soldiers, grimy, dirty, and sunburnt, even their uniform telling of battle and wounds.

### The Scottish Hospital.

At Kroonstadt we were kindly taken over the Scottish Hospital—to-day leaving for home—reported the best in every way, and one did not wonder at such a verdict. It contained about 250 beds, many of them accommodated in huts made of compressed paper, varnished, and looking as comfortable as any ward in England. The theatre was beautifully clean and well appointed—the whole a real tribute to the power of mind over matter, when one reflected for a moment that this embodiment of science was found in the middle of the interminable veldt. Close at hand were a gun and trenches, in which at night the men were doubled, and between 4 and 6 a.m. their numbers still further increased as the Boers were not far off. We slept the night at Kroonstadt and expected to be there some part of the following day, as we heard on arrival that the line had been blown up in three places ahead; however, we started in the forenoon accompanied by two armoured engines, but were not "held up" by the Boers, as we almost hoped, and met with no kind of mishap. We were told that it was the first time the new bridge over the Vaal, just completed, had been crossed.

### Arrival at Pretoria.

As we neared Pretoria, the country became more and more like that of a civilised land. We saw tall chimneys and quite a city of corrugated iron houses at Elangsfontein, half an hour by rail from Johannesburg, and after two hours' more steaming reached our destination—a very pretty town surrounded by hills on which can be seen some of the forts which were to have bidden defiance to the British army. The reddish colour of the earth adds greatly to the beauty of the town, contrasting with the exquisitely vivid green of the willow trees and the profusion of peach blossom which one sees on all hands. Roses and sweet smelling shrubs clamber over the bungalows—there are few storied houses in Pretoria—the shops are fuller than could be expected, though now out of many of the commonest necessaries; the fine Raadzaal and Palace of Justice—the latter commandeered by Lord Roberts for hospital purposes—facing each other in the huge market square, where stand the Dutch Reformed Church and the Dopper Church. The weather, supposed to be that of early spring, is exceedingly warm, and the nights seem hardly less so. We have already made acquaintance with swarms of locusts and first-class dust storms—red dust which soon makes linen khaki colour and bids defiance to all thoughts of real cleanliness in the house; yet we are very glad that our stay is to be here in the capital of the Transvaal.

### The Branch Imperial Yeomanry Hospital at Maitland.

By a Sister at Maitland.

A branch of the Imperial Yeomanry Hospital has now been established at Maitland, about four miles from Cape Town. Its object is twofold—first, as a resting-place for those who have been invalided home, but who often have to wait some time for the hospital ship to take them; and also because cases of acute illness arise, and accidents occur from time to time to yeomen who are serving at the base depôt or the remount camp near. This small hospital contains just over 100 beds: it is rapidly becoming very compact and comfortable. At the same time everything is being organised with a view to its possible increase should the need arise.

### The Wards.

There are two large huts: A, for surgical cases. This hut is subdivided into a ward containing twenty beds for men, a convalescent room, a small ward containing beds for four officers, and an operating room. Hut B contains twenty-two beds reserved for the more acute medical cases. The wards are excellently ventilated and well furnished, and the operation room is fitted with all that is necessary.

### The Beds in Tents.

The rest of the beds are in tents—chiefly marquees—containing six or eight beds each. These tents are double, and all have boarded floors, so that they are very comfortable; indeed, the men often say they are quite the best tents they have seen, and are very contented to be in them. As we get a large proportion of convalescent patients, the tents are filled with those who are able to be out in the fresh air during most of the day. At the back of our camp there are pleasant woods, where wild flowers of many varieties unknown to us at home grow; and in these woods those who are able wander about, and come home laden with flowers, so that our wards are generally gay enough. Among "weeds" are reckoned arum lilies, which grow in such profusion that we can have masses of them for decoration almost daily. There are balconies, too, at the ends of our huts, from which we have delightful views of the sunsets and sunrises over Table Mountain. One large tent is set apart for a recreation tent. It contains a really good piano, large tables where the men can write or play games, and easy chairs. It is well supplied with papers and magazines. The piano, easy chairs, music, and books have been sent by a lady now in Cape Town, who is acting for friends of the Yeomanry at home, and they are all immensely appreciated.

### Visitors and Nurses.

We get a good supply of fresh milk and eggs, and the convalescent patients also fare well, the food being good, abundant, and well cooked. The ordinary fare, too, is often supplemented by gifts from ladies in Cape Town, who drive out, bringing with them light puddings and cakes, jellies, fruit, new-laid eggs, and fresh butter. We always have one Sister on duty, whose employment in the afternoon is especially to escort visitors round, allowing them to distribute flowers, tobacco, papers, &c., to the patients themselves, but reserving the right of giving food, and we never have any difficulty with, nor any attempt at nursing in the wards by, our visitors.

### Tired of Kopjes.

We have already sent off two detachments of patients for England. Most of them were delighted to be "homeward bound." Many were sadly disabled, and others had been ill for months with enteric, dysentery, or malaria; but by the time they left all were sufficiently convalescent to get about, and in the case of the sick the voyage will probably set them up completely. I think I must finish this short account with an anecdote of one of my patients. Some very pretty pictures were sent to the ward, one of them an engraving entitled "At the Foot of Snowdon." In the foreground are some cattle wading, the rest of the picture being the mountain. "That's very nice, Sister; but the kopje spoils it. Soldiers has got tired of kopjes," remarked the man, with a broad Somersetshire accent.

### The Opening of "St. Andrew's House."

Although "St. Andrew's House" Hotel and Club for Nurses has been occupied for some months past, the formal opening was postponed until last Saturday, when everything was finished, and a reception was held for nurses and friends. The proceedings were quite informal; there was no speechifying, and after writing one's name in the Visitors' Book in the entrance, one was free to wander over the house.

### The Reception.

Visitors were received in the smoking lounge by Miss Edith Debenham, and invited to go up in the lift to the top floor, inspect the kitchens, &c., and work their way down to the tea-room, where maid-servants handed round tea and coffee on little trays. There were several nurses in uniform among the guests, and many more were present, but unrecognisable for the occasion, being, as one of the ladies helping Miss Debenham said, "in their best."

Now that the finishing touches have been put, one realises the amount of taste and thought bestowed on even the smallest detail, down to the shape of the bright copper utensils in the kitchen, and the colour of the flowers which decorated the reading room for the occasion. This, by the way, is a most fascinating room. There are good engravings on the walls, which harmonise well with the colouring of the sealing-wax paint and brown paper. A variety of books fill the shelves, among which are many modern novels as well as old standard ones, and a charming set of Shakespeare's plays has a corner to itself. On the table were papers and magazines.

### Over the Tea-cups.

In the tea-room there was plenty of opportunity for chatting, and on all sides one heard enthusiastic praise of the arrangements, and especially of the good taste displayed in the choice of colours, furniture, &c. "It does one good to see anything so pretty," one visitor remarked to another. At a small table two nurses were studying the dainty green prospectus, and were particularly pleased with two "extras," "use of shampooing apparatus (to include materials required), 6d.; use of telephone, 3d." At another, the cost of living at St. Andrew's House was being discussed.

"The items look small on paper," said a nurse; "but of course they must mount up if you have everything; the actual cost comes to about two guineas a week." One visitor was quite of opinion that only nurses with private means could afford the luxury of St. Andrew's; while another rejoined that the prices charged were very low, considering all that is included, and that everything is of the most up-to-date description, adding that the charges had been calculated as low as is possible, consistently with the idea of making the institution a self-supporting one.

### Presentations.

Royal Hants County Hospital, Winchester.—On her resignation of the post of matron, Miss Mocatta was presented by the nursing staff with a case of fish and dessert knives and a salver, subscribed for by forty-three of the sisters and nurses in token of their affectionate esteem. From the hospital servants Miss Mocatta has received a silver-mounted biscuit box and a serviette ring.

Victoria Infirmary, Glasgow.—Miss Foulkes, night superintendent at the Victoria Infirmary, Glasgow, has been presented with a handsome silver kettle and stand, and three fern-pots, by the nursing staff as an expression of their appreciation of her work during the last five years. Miss Foulkes is leaving the hospital, and will be much missed.

### The Nurse in Hot Climates.

By EDWARD HENDERSON, M.D., F.R.C.S. Edin., late Surgeon, General Hospital, Shanghai.

(*Continued from page* 43.)

*The Cold Bath, &c.*—In the treatment of heat-stroke the reduction of temperature by the use of cold water is chiefly of importance, and must now occupy our attention. The bathing or douching of the patient is generally done under the personal superintendence of the doctor in charge or his assistant; and in England the services of the nurse will seldom be called into requisition unless under some specific direction of the kind. In the East, however, the position is not at all an impossible one, and the nurse should be prepared to act in an emergency. The writer can recall more than one case of heat-apoplexy in which, in the absence of a doctor, life was apparently sacrificed to delay in treatment. If along with a high temperature (106° Fahr.) there is delirium or any loss of consciousness the case is urgent. As a general direction, the nurse may make the necessary preparation for the use of cold water when her patient's temperature has reached 105°, and at 106° treatment in one or other of the ways to be described presently may be begun. It is, of course, understood that temperatures are taken in the mouth, only when the state of the patient admits of this being done quite satisfactorily; in heat-apoplexy the rectum is usually the only place in which accurate observations can be made. When a bath is used, the water, between 80° and 90° to begin with, may need to be reduced considerably below this—to 60° or 70°; and it is obvious that in hot climates to get water at these temperatures it will almost invariably be necessary to add ice. In the treatment of these cases of hyperpyrexia, the application of cold, as advised by Manson, should be discontinued before the temperature has been greatly reduced. This should be done, and the patient removed from the bath, when the thermometer registers 102° in the rectum, in cases in which the temperature has not previously been over 106°; at 104° in the rectum in others in which higher temperatures than 106° have been reached. Sudden heart failure is a risk always more or less present in these cases, and one which must be carefully guarded against. Stimulants are often needed, and there is nothing in the high temperature of the body to contraindicate their use; to patients who are unable to swallow they must be given by the bowel. If, as often happens, death from failure of respiration is threatened, recourse must be had to artificial respiration which should always have a full trial before the case is abandoned as hopeless. It is by no means an easy task to lift a patient in and out of a bath who is semi-conscious and unable to help himself, and the necessary assistance cannot always be obtained even in hospitals; this difficulty to some extent limits the use of the cold bath. In an emergency, as in the case of soldiers on the march or cases treated in the open air, douching with cold water can be conveniently carried out with the patient lying on an inclined stretcher, in the way practised and advised by Chandler. The principle is that of the Thomas couch, a principle easily applied, and one to be recommended for use in hospitals. If the couch is made entirely of bamboo, it is easily carried about from one ward or verandah to another by a single attendant. The bamboo settees so largely used by the natives of China to sit and sleep on in the open air are easily converted to this use, for, beyond the removal of the cross-bar at the lower end, to make way for a bucket put there to catch the water as it runs away, very little is needed in the way of further adaptation. The top, or seat, of these couches is made of open bamboo work, through the meshes of which the water poured over the patient escapes readily. To catch and to conduct this water to the bucket a receiver, shaped like a trough and made of some waterproof material such as American oilcloth, is needed; this is fastened below the seat by tapes tied along the sides of the couch, and it should slope towards the lower—the bucket—end. The patient under treatment is stripped and covered with a sheet, his position on the couch making it an easy matter for the attendants to take temperatures, to give stimulants, or to make any direct application of ice, &c., which may be thought necessary. If the case is a troublesome one the bucket may need to be emptied and replaced many times; but in the East, where labour of the kind is so easily obtained, such a detail is immaterial. When there is no ice, and the temperature of the water cannot be sufficiently reduced, a punkah might be used to hasten evaporation from the wet sheet. When a suitable bath and the necessary assistance cannot be got, recourse must be had to cold sponging, and the application of cloths wrung out of cold water. This last plan is usually the first to be tried in the hyperpyrexia of young children; the effect is in nearly every case sufficient, and is easily regulated by the temperature of the water, and the more or less frequent changing of the wetted cloths.

*Mosquito Nets and Mosquito Houses.*—In what was said about the punkah reference was made to mosquito nets, but these useful appliances cannot be dismissed with casual mention only. Protection against mosquitoes is one of the most important of the special arrangements made in connection with the patient's bed in hot climates, and as such is a matter which directly concerns the nurse. The bite of a mosquito is rarely followed by any more serious immediate consequences than that of a limited area of surface inflammation attended by itching of a more or less annoying kind; but the effect varies with varying local conditions, and a good deal depends on the situation of the bite and individual susceptibility to the poison. Tolerance is in most cases to a great extent established after a few years' exposure; indeed, the bite on the skin of an old resident shows often as little more than a red speck, while itching may be altogether absent. If the local effect were all that had to be considered the use of the mosquito net, however desirable, could scarcely be described as one of the most important details in the arrangement of the patient's bed. At the present date, however, when the mosquito has been clearly shown to be the principal, if not the sole, agent in the dissemination of the blood parasite on which malarial fever in man depends, our attitude as regards protection against the attacks of the insect has altogether changed, and what was at one time regarded as expedient only has now become a duty which ought never to be neglected or imperfectly discharged. The fact that it is only mosquitoes of a certain kind, and on the whole of limited distribution, which are to be dreaded in this way scarcely affects the question, as in any particular case the entire absence of these would need to be demonstrated before protection could be safely spoken of as unimportant so far as malaria is concerned. Apart from their connection with malaria, mosquitoes are undoubtedly a source of considerable annoyance to all who are exposed to their attacks; indeed, in really hot nights abroad, when sleep comes with difficulty to everyone, the humming noise which these insects make is often alone sufficient to disturb the repose of a man in perfect health. For this reason, if for no other, the nurse must pay particular attention to the arrangement made for the protection of the patient's bed.

(*To be continued.*)

### "Nurses in Council" and Industrial Law.

THE debate on "Industrial Laws as they concern Nurses," which was held at the Trained Nurses' Club, 12 Buckingham Street, on Friday last, proved of extreme interest. Miss Amy Hughes, of The Nurses' Co-operation, presided, and there was a good attendance.

### APPEAL TO NURSES BY A BISHOP'S WIFE.

Mrs. Talbot made a sympathetic appeal to nurses to help the work of the Industrial Law Committee by teaching the women and child workers in workshops and factories to help themselves by taking advantage of the protection now provided for them by law, but which, from ignorance and timidity, they often failed to use. She pointed out that the law was entirely on the side of the workers, and that the evil conditions under which so many laboured were the result of its evasion by employers, while terror-struck girls and women submitted helplessly to the wrong, lest their struggle for a living should be made greater. The Industrial Law Committee did not combat work, not even hard work, but the dangerous and shameful conditions under which such work was often carried on. "The divine virtue of discontent" needed to be aroused in women in order that they should no longer be contented to acquiesce in evil conditions, but insist on their right to the protection afforded to them by the law. Nurses, who were felt by the poor to be their truest friends, had the most valuable opportunities for helping in this respect, and to do so they needed to know what the law was. With regard to the difficulty experienced in securing evidence of wrong conditions, Mrs. Talbot pointed out that the committee had established an "indemnity fund," for the benefit of those women and children who might lose their employment through giving information as to infringement of the law, a safeguard which it was hoped would encourage those who suffered under such infringement to make known the facts of their case.

### THE VIEWS OF A LADY SANITARY INSPECTOR.

Miss de Chaumont, sanitary inspector for Kensington, gave a clear and comprehensive summary of the principal provisions of such Acts as the Factories and Workshops Act, the Shop Hours and Public Health Acts, showing the distinctions drawn between men's, women's and children's labour, and the ages at which the workers came under the different restrictions; the cubic space provided for by its clauses, and the means for regulating temperature, &c. She explained the powers of the inspector with regard to the provision of proper sanitary conveniences, the adequate ventilation of work-rooms, the fencing of machinery, and the working of laundries. Miss de Chaumont spoke of the restrictions as to hours of work in various employments; of overcrowding in houses, under the Public Health Act, and of nuisances; provisions as to drinking water, and all the various ways in which the law can step in to protect the life and health of women and child workers; giving most valuable information for nurses to act upon when in their daily work they find suffering and disablement arising from evasions of the industrial law. Nurses need not complain themselves, for their position was naturally a delicate one, and they had to avoid any appearance of "spying"; but they could teach the people to complain, tell them the proper quarters to which to send their complaints, and by advice and encouragement help their poor patients and their friends to a knowledge of the means actually at their doors for availing themselves of the protection intended for their use.

### MISS NIGHTINGALE'S ASPIRATION.

Miss Hughes, in thanking Mrs. Talbot and Miss de Chaumont for their addresses, urged those present to take every advantage of their opportunities as nurses to help other working women in the directions pointed out, when they would be fulfilling Miss Nightingale's own strongly-expressed aspiration that nurses should as "health missioners" carry far and wide a knowledge of the laws of sanitation.

It may be added, for the benefit of nurses generally, that information sent to the office of the Industrial Law Committee, 5 Palmer Street, Westminster, is sifted and sent on to the proper authorities for dealing with any complaints that may arise, and that particulars as to the work of the committee can be had on application to the secretary, Miss Boileau.

### Death in our Ranks.

A CORRESPONDENT writes: Many of your readers will doubtless recognise the name of Nurse Sarah Ann Markham, who died suddenly, while nursing at Folkestone, at her post early in the morning of St. Luke's Day. She had just finished her day's work and was preparing for her night's rest, when she fell, struck down all at once, and though doctor and nurse were summoned, and everything possible was done, she never rallied, but passed away painlessly an hour after the seizure, without regaining consciousness. Nurse Markham was for some years a member of the Guild of St. Barnabas, she was also a life member of the Royal British Nurses' Association, and their motto, "Steadfast and True," was strictly adhered to through her life. She was steadfast, true, and faithful in all her work; conscientious, and never failing in her duty. As one who lived and worked with her for nearly ten months, in daily and hourly contact, I feel that I have lost not only a friend, but also the straightest, truest, sincerest example. She was born on May 30th, 1851. She was trained at Middlesex and Reading Hospitals; and for some time she was one of the Stoke-on-Trent Nurses.

WE regret to hear of the death of Nurse Julia Catherine Wooldridge, Superintendent of Children's Department, Barnhill Poorhouse, Glasgow, from enteric fever after a short illness, at the age of 35. She received her training in the Salford Union Infirmary, afterwards acting as charge nurse two years, at Craiglockhart, Edinburgh, superintendent of Nurses for four and a half years at Smithston Infirmary, Greenock, and was connected with the Higginbotham private staff in Glasgow for four years. She was highly esteemed by all who came in contact with her.

### Hints to Nurses.

### FADED LEAVES.

Now that autumn, with all its beautiful changes of colour, is really here, I should like to offer a suggestion to those nurses whose work calls them into the country. In the hedgerows and old walls, &c., are many little hardy ferns, trails of brilliantly-tinted, tiny leaves, &c. Gather these on a nice bright dry day, press them in your ordinary writing-paper or in your blotting-pad, and in a few weeks they will be fit for use. *Then*, when your trying "private patient" (young man, young woman, child, or even an elderly body) is convalescing, bring your leaves out, with a pot of gum or some cold mucilage starch, and set your patient to work. The prettiest little cards can be made, and are always very greatly appreciated by friends. The gathering of the leaves is a never-failing source of interest to both nurse and patient; it is something to have an object for your daily walk. You and your patient will both benefit, and there will be pretty little mementoes which your friends will treasure more than you imagine. A postage-stamp photo of the sender, unmounted, may be stuck on to a good-sized card, and a decoration of flowers and leaves placed around. The effect is very good. It is only a small thing to do, but I thought others might like to hear of it, and this is the best time to begin collecting. If kept dry, the pressed leaves will last for years, and can be used any time.

### Appointments.

ROYAL INFIRMARY, LIVERPOOL.—Miss Elizabeth Mary Jones has been appointed lady superintendent and matron. She was trained at the Hospital for Sick Children, Pendlebury, and the Royal Infirmary, Liverpool. She has since successively been sister of medical and surgical wards, night superintendent, and assistant lady superintendent and matron in the same institution.

REIGATE NEW ISOLATION HOSPITAL.—Miss Lucy Amcoats has been appointed matron. She was trained at the Royal Infirmary, Liverpool, and has since been matron for thirteen years at Douglas Isolation Hospital, and matron for one and a half years at Croydon Rural District Council Isolation Hospital.

WHITECHAPEL INFIRMARY.—Miss Eleanor Taylor has been appointed assistant matron. She was trained for three years at Shoreham Infirmary, Sussex, and remained afterwards for two years as assistant superintendent nurse. She has since been night sister at the East Suffolk Hospital Ipswich.

BIRMINGHAM GENERAL HOSPITAL.—Miss H. Hannath has been appointed night superintendent. She was trained at King's College Hospital, and has since been sister in charge of sick-nurses and lecturer on sick-room cookery at the London Hospital, night superintendent at the Bristol Royal Infirmary, and superintendent nurse at Eastville Union Hospital, Bristol.

### Minor Appointments.

DISTRICT HOSPITAL, WATFORD.—Miss Mary McKinnell has been appointed staff nurse. She was trained at East Dulwich Infirmary.

DEVONPORT INFIRMARY "FORD."—Miss Ada M. Coleman has been appointed charge nurse. She was trained at Mile End Infirmary, and has since been doing private nursing at Southend, Essex.

MILITARY HOSPITAL, ATHENS.—Miss Alithea Williamson has been appointed head sister at the Military Hospital Athens. She was trained at the North Staffordshire Infirmary and Eye Hospital.

BOOTLE BOROUGH HOSPITAL, LIVERPOOL.—Miss J. M. Oliver has been appointed night sister. She was trained at the Royal Infirmary, Manchester, and has held the post of charge nurse at the Brook Hospital, Shooter's Hill.

BARROW-IN-FURNESS HOSPITAL.—Miss Elinor Hulme has been appointed charge nurse. She was trained at Noble's Hospital, Douglas, Isle of Man, where she has also been charge nurse. She has since been sister at Southport Infirmary, and has done some private nursing.

CROMER DISTRICT NURSING ASSOCIATION.—Miss M. A. West has been appointed district nurse. She was trained at Dr. Barnardo's Hospital for Children, British Lying-in, and Poole Hospital. She was on the Fakenham nursing staff for sixteen years, and has been engaged in district work for some years.

THE ROYAL HOSPITAL FOR CHILDREN AND WOMEN, BRISTOL.—Miss Constance J. Morgan has been appointed night sister. She was trained at East Dulwich Infirmary and was promoted to the post of sister. Subsequently, she was head nurse at the Children's Hospital, Sydenham, and charge nurse at the South Western Fever Hospital, Stockwell. Miss Morgan received her midwifery training at the East End Mothers' Home, Stepney, and holds the L.O.S. certificate.

### Everybody's Opinion.

### NURSING AT KIMBERLEY.

"A LATE ARMY NURSING SISTER" writes from Kimberley, South Africa, under date of September 30th: My comments on the article on p. 241 of the NURSING MIRROR, August 4th, 1900, are naturally late. But for the sake of truth, and in the interest of your South African readers, you will perhaps print the following:—I also was in Kimberley during the siege; have lived here, in fact, for three years, the first two months of which were spent as a member of Sister Henrietta's nursing staff. The late Mr. Labrane, chief engineer of the De Beers Company, was not killed by a bursting shell, but buried and suffocated under the collapsing wall of the reading-room in the Grand Hotel, next door to the room where the shell exploded. As the deceased gentleman was universally respected, the authorities wanted to give all his friends a chance to pay him the last honour, and therefore sent a white flag to the Boer commandant, requesting that the firing might cease during the funeral. There was a ready and courteous assent, and I remember well that, between 4 and 6 p.m. on the day of the funeral, the guns were silent. As this was in the early (South African) autumn, it was not dark at the time of the funeral.

### ASYLUMS BOARD NURSES.

"A NURSE IN THE COLONIES" writes: In THE HOSPITAL of June 30 you quote Dr. Goodall, medical superintendent of the Eastern Hospital, who says that "when the charge nurses have received instruction on nursing in a fever hospital, and have served long enough to be entitled to a testimonial, off they go." Speaking from personal experience, I do not think that Dr. Goodall is quite fair to the charge nurses. When a nurse receives a form to fill in previous to her being engaged as a charge nurse under the Metropolitan Asylums Board, there is no mention of night duty. As a charge nurse's duties do not generally include night duty this is a serious grievance, and has been the cause of a great number of nurses leaving, some at the end of their first month, some the day after their arrival. Those nurses who would do anything for peace, and who dislike changes, have accepted the night duty, but have found fault with the arrangements made for the night charge nurses. There is no separate dining-room, all their meals being taken with the assistant nurses. There are several untrained charge nurses who do not go on night duty, so that a nurse who has had five or six years', or in some cases longer, experience in all branches of nursing, finds herself taking orders for the night from an untrained woman. Many of the nurses applying for posts as charge nurses under the Metropolitan Asylums Board do not wish only for fever training. Often they apply because after a year or two of private nursing they desire to get back into hospital again, and would be very pleased to stay there if circumstances would permit. The ambulance grievance is the great complaint of the day charges. A charge nurse is given a ward, with one or two assistant nurses, according to the size of the ward. The majority of the patients are children. The charge nurse and her assistants go on duty together. There is none of the nice feeling which prompts the probationer to get the ward straight before sister comes on duty. Sometimes, when a charge nurse has only one assistant nurse, in the midst of work, at any hour, the ambulance nurse will send for the assistant without the charge nurse being asked if she can spare her, and, as the majority of the assistant nurses seem to prefer riding in a van to ward work, there is no redress. It may be dinner, lunch, or tea time, or the time of the doctor's visit; nothing alters the fact that the assistant is wanted for ambulance duty, and off she goes. This is very annoying to a nurse who likes everything done at the proper time, and to have her ward at all times neat and tidy. I do not see why the Metropolitan Asylums Board should not be able to keep their charge nurses. The salary is good, so is the off-duty time; Dr. Goodall is an excellent medical superintendent, and a kind man both to patients and nurses; the food is good and abundant, the bedrooms are comfortable, and the sitting-rooms nicely furnished. Most nurses engaged in fever hospitals visit their friends just the same after thoroughly disinfecting themselves, and any one nursing in a fever hospital for a single day runs as much risk of infection as she does in a dozen years.

### Echoes from the Outside World.

### AN OPEN LETTER TO A HOSPITAL NURSE.

OWING probably to the early age at which she was called to the throne, a special interest has always attached to the doings of the young Queen of Holland, and you may like to hear the latest details of her engagement. The bridegroom's name, as you know, is Henry; but with much tact Queen Wilhelmina, knowing that it would please her subjects, in writing of her fiancé alluded to him as Hendrik, instead of Heinrich. The official announcement—beginning "My heart prompts me to make known by my own hand to my people, the Dutch nation, . . . the news of my betrothal"—was very simple and nice. As in the case of our own Queen, it was the lady who had to suggest to the gentleman that any advances he might make would not be altogether distasteful to her, because it would be against royal etiquette for a prince of inferior position to woo a queen. Therefore, accompanied by her mother, Queen Wilhelmina went to the house of her aunt, the Countess of Arnberg, from Saturday to Monday, the hostess having also invited Duke Henry of Mecklenburg-Schwerin to come and stay with her. There the proposal was made. The romance, for such it now appears to be, began in the summer, when the young couple were introduced at Berlin. They mutually liked each other even at the first meeting, so much so that when a dinner party was given where several "eligibles" were to be present for her benefit, it is said that the young Queen pleaded a cold as an excuse for not appearing, as she had already made up her mind where she would bestow her heart and hand. Then the holiday of the two Queens was spent at Schwarzburg, and the young couple frequently met, the Queen talking in Low German, as the Prince did not know Dutch. However, he will probably speedily learn it, and meantime he rides out daily in Amsterdam with his bride-elect, who is said to look radiant. He is a finely-built, well set-up young fellow, has fair hair and blue eyes. The German Emperor seems to be delighted with the betrothal, and sent Duke Henry a long congratulatory telegram.

I WONDER if many of you found time to read Sir Redvers Buller's speech at Maritzburg? It appears to throw a light on much in the conduct of the war which has seemed inexplicable, and it also shows that a soldier can be brave in words as well as in deeds. The General said he had received many letters of abuse, criticising his plans, but if he had sat down in Natal and waited for his army to arrive—at the time he landed at the Cape most of his men and horses were on the high seas or had not yet left England—the Boers would undoubtedly have occupied the whole of the Colony. Sir Evelyn Wood had telegraphed that he was willing to come out and serve under him, and he thought he had never been so much tempted in his life as he was to accept that offer. But he had begun to look upon the work to be done in Natal as almost a forlorn hope, and he did not see that it would be right to take advantage of Sir Evelyn's generosity and let him run the risk. He had been in great anxiety over the failure to relieve Ladysmith in December—a failure which lost him the command, "and," he added, "rightly so." General Buller went on to say that his was the most gallant army that any officer had ever commanded, and that he had received the greatest assistance from the Natal colonists. He denied that he had ever been told to act this or that way for political reasons, the Government having always given him a perfectly free hand. It will not be long now before General Buller arrives in England, as he sailed from Cape Town last Wednesday, and I hope that the English nation will receive him as he deserves and remember that he succeeded in an almost Herculean task, namely, the relief of Ladysmith. Meanwhile the C.I.V.s seem likely to be almost killed with kindness when they arrive on Saturday. The relatives of the men have, in many cases, been offered seats along the route by householders, but on the other hand several shopkeepers and those who have offices along the route have failed to learn the lesson which the Diamond Jubilee should have taught them. It is possible to be too over-reaching and £200 for a shop front, or 60 guineas for a first floor to seat 12 persons, are amongst the prices advertised. I fancy that even the most patriotic will kick at such fancy sums as these.

PRIOR to General Buller, another of the figures of the South African war left its shores, probably never to return. I allude to Mr. Kruger. His departure may not have been either dignified or heroic, but at least it was consistent. It was only to be expected that the man who drew the Orange Free State into his quarrel, not because it had anything against Great Britain, but to strengthen his hand; who sent his men to fight whilst he stopped in the background; who, collecting all the money he could lay his hands upon, fled from his capital, leaving his wife behind, and, in terror of being captured, travelled by night, should embark on board the *Gelderland* at five o'clock in the morning for fear his angry burghers should frustrate his cowardly plans. All this is no more than one would anticipate, but that any person should be found to propose that Dublin should present him with the freedom of the city, or that efforts should be made to render his progress through France a series of ovations, is much more difficult to understand. Happily, the protest of a well-known French writer against any such ill-timed demonstration indicates that some Frenchmen are still sensible. Those who talk about offering him houses and hospitality, unless it is all to be on the gratis system, should remember that, although the ex-President of the Transvaal took away with him large sums of money, even the smallest debts he contracted have remained unpaid, and his late officials are trying in vain to get the Embassy to honour the cheques given them for salaries last May. They ask in vain. The Embassy officials maintain that there is no money in hand, and say that they themselves are poor and needy.

WOMEN as a rule do not make good prophets. The feminine attribute of exaggeration is too apt to creep into the prophetic utterances, so that the future is either too rosy or too gloomy according to the desire of the soothsayer. But a prophetess who has just uttered dark sayings in South Africa has quite beaten the record for piling up horrors, irrespective of probabilities. "Olive Schreiner" has been seized with a desire to warn England of forthcoming events, and she predicts its early downfall. This is nothing fresh, but the details have the attraction of novelty. The author of "The Story of an African Farm" sees a vision "The Russian, French, or German troops are upon the soil of England; Englishmen are gathering to defend Richmond Hill or Hampstead Heath, the tramp of foreign soldiers is heard in the streets of London, and the ground is wet at Marble Arch and Hyde Park Corner with the blood of Englishmen. Then when that day comes, let England remember South Africa." But this is not all. Our souls, too, will be burdened with remorse, for "Time will show that it would have been cheaper to have paid a million to each Boer than to have killed them." I did not know that the war had been upon a question of compensation, but perhaps the suggestion is that the Boers having declared war upon us, we should have paid large sums of money to each unit of the Transvaal Army so as to secure peace. This view of the case had not struck most of us before.

### For Reading to the Sick.

. . . . WHO best
Can suffer, best can do ; best reign, who first
Well hath obeyed.—*Milton.*

THY prayer shall be fulfilled ; but how ?
His thoughts are not as thine,
Whilst thou wouldst only weep, and bow,
He saith, "Arise and shine !"
Thy thoughts were all of grief and night,
But His of boundless joy and light.
Thy Father reigns supreme above :
The glory of His name
Is Grace and Wisdom, Truth and Love,
His will must be the same.
And thou hast asked all joys in one
In whispering forth, "Thy Will be done."

—*F. R. H.*

### Reading.

WE know not exactly how low the least degree of obedience is which will bring a man to heaven ; but this we are quite sure of, that he who aims no higher will be sure to fall short even of that, and that he who goes farthest beyond it will be most blessed.—*Keble.*

THAT God has circumscribed our life may add a peculiar element of trial, but often it defines our way and cuts off many tempting possibilities that perplex the free and the strong ; whilst it leaves intact the whole body of spiritual reality, with the Beatitude thereon, "that if we know these things, happy are we if we do them." We know that God orders the lot; and to meet it with the energies it requires and permits, neither more nor less—to fill it at every available point with the light and action of an earnest and spiritually inventive mind, though its scene be no wider than a sick chamber, and its action narrowed to patient suffering and gentle, cheerful words, and all the light it can emit the thankful quiet of a trustful eye—without chafing as though God had misjudged our sphere, and placed us wrong, and did not know where we could best serve Him—this is what, in that condition, we *have to do.—J. H. Thom.*

THE crosses of the present moment always bring their own special grace and consequent comfort with them ; we see the hand of God in them when it is laid upon us. But the crosses of anxious foreboding are seen out of the dispensation of God ; we see them without grace to bear them ; we see them indeed through a faithless spirit which banishes grace. So, everything in them is bitter and unendurable ; all seems dark and helpless. Let us throw self aside ; no more self-interest, and then God's will, unfolding every moment in everything, will console us also every moment for all that He shall do around us, or within us, for our discipline.—*Fénelon.*

THE little sharp vexations
And briars that catch and fret,
Why not take all to the Helper
Who has never failed us yet ?
Tell Him about the heartache,
And tell Him the longings too ;
Tell Him the baffled purpose
When we scarce know what to do.
Then, leaving all our weakness
With One divinely strong,
Forget that we bore the burden
And carry away the song !

—*Phillips Brooks.*

### To Nurses.

WE invite contributions from any of our readers, and shall be glad to pay for "Notes on News from the Nursing World," or for articles describing nursing experiences, or dealing with any nursing question from an original point of view. The minimum payment for contributions is 5s., but we welcome interesting contributions of a column, or a page, in length. It may be added that notices of entertainments, presentations, and deaths are not paid for, but, of course, we are always glad to receive them. All rejected manuscripts are returned in due course, and all payments for manuscripts used are made as early as possible at the beginning of each quarter.

### Notes and Queries.

**The Editor is always willing to answer in this column, without any fee, all reasonable questions, as soon as possible.**
**But the following rules must be carefully observed :—**
**1. Every communication must be accompanied by the name and address of the writer.**
**2. The question must always bear upon nursing, directly or indirectly.**
**If an answer is required by letter a fee of half-a-crown must be enclosed with the note containing the inquiry.**

*Massage Fees.*

(40) After nursing a case of hypochondriasis for three weeks the doctor ordered massage. Am I entitled to charge an extra fee for doing it ? I am working on my own account.—*A. L. J.*

Certainly. But why leave such a loophole for unpleasantness to creep in ? Have your terms printed on your card, and distinctly draw your patients' attention to the fact of extra fees for extra service. Set the matter right at once by clearly stating your requirements as to remuneration.

*Lecturer.*

(41) In order to obtain a post of County Council Lecturer to whom should application be made ?—*J. G.*

For particulars of the certificate of the Sanitary Inspectors' Examination Board, apply Hon. Secretary, 1 Adelaide Buildings, London Bridge ; for those of the certificate of the Sanitary Institute, apply Secretary, Margaret Street, London, W. For the diploma of the National Health Society, apply Secretary, 53 Berners Street, Oxford Street, W.

*Post.*

(42) Will you kindly tell me to whom I should apply for a post in the Holland Institute, Nice ; and (2) also for a Government appointment in India ?—*Matron.*

The Nice Nursing Institute (formerly the Holland Institute), Villa Soleil, Avenue Thiers, Nice. (2) The Secretary, The Indian Nursing Service, India Office, St. James, S.W.

*Epileptic.*

(43) I should like to know if there is any home or settlement for an epileptic woman able to do a little work.—*Nurse F.*

The National Society for the Employment of Epileptics, Chalfont St. Peters (office, 12 Buckingham Street, Strand, W.C.) ; the Home for Epileptics, Maghull, near Liverpool ; and the Meath Home of Comfort for Epileptic Women and Girls, Godalming, are the three public institutions for this class of sufferers.

*Supplementary Training.*

(44) Would you tell me which hospital will take a nurse with two years' general training, and give her, at the end of one year's instruction, a three years' certificate ?—*Nellie.*

No institution offers such liberal terms as you ask. The Victoria Park Hospital, London, E., will give such a certificate after two years' work, provided the matron is satisfied with the preliminary instruction.

*Lectures.*

(45) Will you kindly tell me where in London I could attend hospital or nursing lectures ? I hope later to enter a hospital for training, and I am most anxious to increase my knowledge of the subject.—*E. S.*

You might write to the Secretary of the Trained Nurses' Club, 12 Buckingham Street, Strand, W.C., but such lectures will not be of much service. The hospital training is the thing.

*Abroad.*

(46) Can you help me to get information as to a good nursing home in Australia, Colombo, or Canada, with particulars of salary and home comforts ?—*H. S.*

The Melbourne Nurses' Home, 6 Parliament Place, Melbourne, Lady Superintendent, Miss Glover. Nurses (only certificated nurses employed) take their own fees, which are from £2 2s. weekly. The Ceylon Nurses' Association, Lady Superintendent, Miss Ramsay. The Victorian Order of Nurses for Canada, 578 Somerset Street, Ottawa, Ontario. Write fully to the lady superintendents for advice before risking anything.

*Bust.*

(47) 1. Is there any means of developing a bust ? My patient is thin and flat-chested, and thinks that if her figure were well developed, it would protect her lungs. Would rubbing or massage be of service ? 2. Can you tell me anything about the Co-operative Society ?—*Nurse N. C.*

1. Three things are necessary to develop a good chest. First, by suitable physical exercises in respiration to increase the lung capacity ; secondly, by exercise to cultivate the muscles ; and lastly, by improving the general health and diet, to put on flesh. 2. The Nurses' Co-operation is the largest nurses' association in London. It is managed entirely for nurses by nurses, and every member is highly qualified. Full particulars from the Secretary, 8 New Cavendish Street, W.

*Baths for Rheumatism.*

(43) Will you kindly tell me if there is any good hydropathic establishment for the treatment of rheumatism by baths in Droitwich, Bristol, &c. ? I am a nurse, and have had to give up my work here, as rheumatism cripples me. I cannot afford great expense, but I should like to try bath treatment.—*Crippled.*

The baths at Droitwich are very efficacious in some cases of rheumatism. If it be desirable to avoid unnecessary expense, you can obtain a cheap lodging in the town, and attend the baths for treatment. Write to Dr. Roden, the Brine Baths, Droitwich, for full particulars.

### Standard Books of Reference.

"The Nursing Profession : How and Where to Train." 2s. net ; post free, 2s. 4d.
"The Nurses' Dictionary of Medical Terms." 2s.
"Burdett's Series of Nursing Text-Books." 1s. each.
"A Handbook for Nurses." (Illustrated.) 5s.
"Nursing : Its Theory and Practice." New Edition. 3s. 6d.
"Helps in Sickness and to Health." Fifteenth Thousand. 5s.
"The Physiological Feeding of Infants." 1s.
"The Physiological Nursery Chart." 1s. ; post free, 1s. 3d.

All these are published by THE SCIENTIFIC PRESS, Ltd., and may be obtained through any bookseller or direct from the publishers, 28 and 29 Southampton Street, London, W.C.

### Travel Notes.

By Our Travelling Correspondent.

### LX.—ON WINTERING ABROAD.

At the beginning of the winter season I like to hang out a few signals to warn the inexperienced against some dangers that beset the path of those who either from choice or necessity spend their winter months in a foreign country. It is unavoidable that I must necessarily repeat much that I have said before, but I hope that the kindly readers who studied my former articles will bear with their repetition for the sake of those who have not hitherto perused my well-meant lectures on the necessity of much thought and wary preparations for a winter sojourn abroad.

### The Selection of Climate.

I take it for granted that no one (if in ill-health) would dream of choosing any resort for themselves without most careful consultation with their medical adviser. It was formerly the received axiom that all sufferers from pulmonary complaints should winter in mild warm climates, and go out only on fine days for a few hours, carefully avoiding morning and evening temperatures. Now all this is changed; our phthisical friends are sent very frequently to extremely cold places, and they are out as long as possible each day. Then there is a class of semi-invalids who, being in an anæmic condition, are simply required to live in the open air and absorb sunlight, all day and every day. This cannot be done satisfactorily in England—we have so little winter sun; and it is often obscured by mist to such a degree that our Continental neighbours would hardly recognise it as sun at all. The number of those who winter abroad is largely on the increase, and chiefly I believe because of our lack of sun and consequent enforced confinement to the house. Moreover, living abroad is cheap (always provided you know how to manage), and I am constantly helping overworked nurses and others suffering from nervous prostration, &c., to spend a few months in Italy, Southern France, or the Riviera, and thus laying up a store of renewed health for further exertions.

### Northern and Central Italy.

Those with slender purses have until lately thought of Italy as too distant a spot, and too expensive to be visited except by the wealthy. This is a mistake, and I hope to show that the boundless pleasures of Italian travel are within the reach of most.

I purpose this winter to take you with me in spirit about the old towns of Northern Italy and Rome, and once in a way to give you a paper on the art treasures of that fascinating land. Many nurses who propose to take three months' rest would do well to consider the question of spending that three months somewhere in the South, and I feel sure that the small expense of the undertaking, if managed according to my ideas, will astonish them. I shall not enter into all these details in my papers, because space is so limited, but I am always happy to answer any questions and give any advice.

### Unavoidable Expense.

No amount of ingenious contrivance can possibly mitigate the heavy initial expense of a long journey. The ticket to Rome, second class, by the cheapest route is £6 6s.; so that £12 12s. must be set aside for the railway. But once there, living may be managed very cheaply by those who can speak the language at all, and if not, board and lodging can be had in Pensions reasonably. Naturally this kind of living would not be suitable for invalids, but I must ask you to use common-sense in applying my remarks and advice only to suitable cases and in suitable circumstances.

Rome is to many a more desirable city than any other on the Continent, but I should not advise any to go there who have not a marked taste for art in one or other of its branches. If you do not care for sculpture or old masters, or mediæval architecture or Roman remains, then do not go to Rome; for though there is fine scenery round, many excursions to be made, and fashionable society to be encountered on the Pincio, I do not think it is a place for those who are devoid of artistic instincts. Florence or Venice, though both of them wealthy mines of art, are full of other interests also. I feel sure that many of the English who crowd the gay streets of Florence, having once satisfied their consciences by a visit to the Uffizzi and the Pitti, never trouble themselves again to study the vast stores of art gathered together in the city of lilies.

### Relative Merits of Italy and the South of France.

Italy is a country full of charm, and nowhere, I think, are there such interesting cities where one may study so engrossingly the long-buried past, with its history so thronged with romantic interests; but if your tastes do not lie this way, and you prefer splendid scenery, then go rather to the land of Southern France, where, if you are a good walker or bicyclist, you may with very little expense feast your eyes upon new beauties every day. Roughly speaking, I consider Southern France the country for the lover of scenery, and Italy for the lover of history, art, and romance.

### The Cheapest Way of Living.

If you can speak Italian, by all means take a couple of furnished rooms and cater for yourself, engaging the services of an "occasional" woman to clean you up every day. Dinner is taken at a moderate restaurant usually in these cases, or they will send it in to you from a *trattoria*. If you are a party of three or four, two portions will be found amply sufficient; whilst one will thoroughly suffice for two, unless your appetites are unusually large.

It is better to go out for dinner, if you are in good health it is more amusing, and you are better catered for. The system of sending in from a *trattoria* answers admirably for a time, and then carelessness creeps in, which compels one to constant changes of restaurants. Besides which it is very amusing to go out in the evenings to dinner and to try different places every few nights, studying the world of Italy as it eats and drinks its modest meals. It is the custom of the country thus to feed away from home, so that ladies in restaurants cause no remark.

Next time I shall talk to you a little of the things needful to take with you, and those which it is judicious to leave behind, and also try to give you the results of my experiences (often painful ones) as to the best kind of sketching and photographic arrangements.

### TRAVEL NOTES AND QUERIES.

Rules in Regard to Correspondence for this Section.—All questioners must use a pseudonym for publication, but the communication must also bear the writer's own name and address as well, which will be regarded as confidential. All such communications to be addressed "Travel Editor, 'Nursing Mirror,' 28 Southampton Street, Strand." No charge will be made for inserting and answering questions in the inquiry column, and all will be answered in rotation as space permits. If an answer by letter is required, a stamped and addressed envelope must be enclosed, together with 2s. 6d., which fee will be devoted to the objects of the "'Hospital' Convalescent Fund." Any inquiries reaching the office after Monday cannot be answered in "The Mirror" of the current week.

Baden-Baden (Enquirer).—Yes, it is sheltered, and therefore not cold in winter; but I should hardly call it a warm place. In the early spring it is a delightful spot; and if you went before the season begins, say the early part of May, charges would be moderate. In the summer it is very hot. I was there in July for a few days, and the heat was excessive. It is a beautiful centre for excursions.

Avignon, &c. (Costabelle).—Avignon, Nîmes, and Arles are all near together, and may be all visited whilst resident in one. It is a good plan to stay at Tarascon, which is central and near to all three. It is difficult to say which is the most interesting; personally, I prefer Avignon, on account of its historical associations. Not far off is Orange, a curious and interesting old town; and in the other direction Aigues-Mortes. This part is but little known to tourists, and is still primitive and unspoilt, with endless subjects of interest for the artist and archæologist.

